# Heart Failure with Reduced Ejection Fraction (HFrEF) and Preserved Ejection Fraction (HFpEF): The Diagnostic Value of Circulating MicroRNAs

**DOI:** 10.3390/cells8121651

**Published:** 2019-12-16

**Authors:** Yei-Tsung Chen, Lee Lee Wong, Oi Wah Liew, Arthur Mark Richards

**Affiliations:** 1 Department of Life Sciences and Institute of Genome Sciences National Yang-Ming University, Taipei 112, Taiwan; 2 Cardiovascular Research Institute, National University Health System, Singapore 117599, Singapore; mdcwll@nus.edu.sg (L.L.W.); mdclow@nus.edu.sg (O.W.L.); arthur_mark_richards@nuhs.edu.sg (A.M.R.); 3 Department of Medicine, Yong Loo Lin School of Medicine, National University of Singapore, Singapore 117597, Singapore; 4 Christchurch Heart Institute, University of Otago, Otago 8014, New Zealand

**Keywords:** heart failure, microRNA, HFrEF, HFpEF

## Abstract

Circulating microRNAs offer attractive potential as epigenetic disease biomarkers by virtue of their biological stability and ready accessibility in liquid biopsies. Numerous clinical cohort studies have revealed unique microRNA profiles in different disease settings, suggesting utility as markers with diagnostic and prognostic applications. Given the complex network of microRNA functions in modulating gene expression and post-transcriptional modifications, the circulating microRNA landscape in disease may reflect pathophysiological status, providing valuable information for delineating distinct subtypes and/or stages of complex diseases. Heart failure (HF) is an increasingly significant global health challenge, imposing major economic liability and health care burden due to high hospitalization, morbidity, and mortality rates. Although HF is defined as a syndrome characterized by symptoms and findings on physical examination, it may be further differentiated based on left ventricular ejection fraction (LVEF) and categorized as HF with reduced ejection fraction (HFrEF) and HF with preserved ejection fraction (HFpEF). The presenting clinical syndromes in HFpEF and HFrEF are similar but mortality differs, being somewhat lower in HFpEF than in HFrEF. However, while HFrEF is responsive to an array of therapies, none has been shown to improve survival in HFpEF. Herein, we review recent HF cohort studies focusing on the distinct microRNA profiles associated with HF subtypes to reveal new insights to underlying mechanisms and explore the possibility of exploiting these differences for diagnostic/prognostic applications.

## 1. Significance of Heart Failure

Heart failure (HF) is a complex clinical syndrome arising from deficient cardiac output that is unable to meet the metabolic needs of the organs and tissues in the body. HF evolves from systolic and/or diastolic contractile dysfunction caused by progressive structural and functional alterations of the heart [1]. Multiple risk factors are strongly associated with the prevalence and incidence of HF, including age, hypertension, obesity, and diabetes, as well as prior cardiovascular conditions such as atrial fibrillation and coronary artery disease especially when complicated by myocardial infarction [2]. HF may be acute or chronic with many patients experiencing recurrent episodes of acute decompensated HF superimposed on a background of chronic HF, wherein the latter comprises the majority of the HF population [1]. Despite substantial therapeutic advances, morbidity, mortality, and hospitalization rates in HF remain high. HF survival has not improved from a decade ago with mortality of approximately 20% and 53% at one and five years from diagnosis, respectively [3]. It is estimated that 17–45% of hospitalized HF patients die within a year of admission and no more than half will survive beyond five years [1,4]. With an aging population worldwide, the incidence and prevalence of HF is rising, compounded by known age-related comorbidities such as type 2 diabetes mellitus and renal impairment, as well as myocardial infarction and hypertension [5,6,7]. The enormous and growing challenge of HF as a significant global health problem requires urgent attention to accelerate disease characterization, understand HF predictors, and to open windows of opportunities for diagnosis/prognosis, prevention, and treatment.

## 2. Prevalence of Heart Failure

The overall prevalence of HF is about 1–2% worldwide with an estimated 37.7 million people affected globally [4,8]. In the United States and European Union, the prevalence of HF varies from 1% to 14% in various cohort studies involving different age groups [2]. Based on data collected between 2011–2014 in the United States, the prevalence of HF in those over 20 years of age is about 6.5 million (2.4%) with 960,000 new cases reported annually [1]. In the United Kingdom, the prevalence of HF is 1.5–1.6% based on a longitudinal study between 2002–2014 [2]. Notably, further analysis revealed a rise in HF incidence by 23% from 2002 to 2014 in the United Kingdom (from 750,127 cases in 2002 to 920,616 cases in 2014) [2]. The trend is similar in Asia where the overall prevalence of HF is around 1–2% but distributed variably (between 0.3 to 6.7%) across different countries [9]. Asian HF patients are younger than those of Caucasian ethnicity. The contributory factors for this difference remain elusive [9]. Asia has a rapidly aging population of about 4.5 billion people (approximately 60% of the world’s population) carrying a high burden of cardiovascular risk. This portends a burgeoning public health challenge in the region mandating urgent research to understand and characterize geographical, social, ethnic, and phenotypic differences in Asian HF. 

## 3. The Challenges of HFrEF and HFpEF

Symptoms of HF include breathlessness, cough, interrupted sleep, exercise intolerance, edema, and fatigue. However, the nonspecificity of these symptoms can confound the diagnosis of HF as they also occur in noncardiac conditions including renal impairment and chronic obstructive pulmonary disease. More specific signs of HF include elevated jugular venous pressure and the displacement of the apical cardiac impulse. However, these signs may be hard to detect and difficult to interpret in patients with comorbidities such as obesity or airway diseases [10]. Thus, the diagnostic work up for HF requires additional tests which may include chest radiography, electrocardiogram, echocardiography, and measurement of circulating levels of B-type natriuretic peptide (BNP) and/or its co-secreted congener, amino terminal proBNP, (NT-proBNP) [1,11]. Accumulating evidence from multiple clinical studies show that BNP and NT-proBNP are the most reliable markers for diagnosis, prognosis, and monitoring of HF [12,13,14,15]. The single commonly accepted cut-off points for BNP and NT-proBNP in non-acute ambulant cases where HF may be suspected, are >35 pg/mL and >125 pg/mL, respectively. Over the last decade, an expanded role for NPs in predicting HF risk and guiding preventive interventions in both the general and at-risk population has been established [16,17,18,19].

Left ventricular ejection fraction (LVEF) is a widely adopted phenotypic parameter to define HF. HF with reduced ejection fraction (HFrEF; EF ≤40%) and HF with preserved ejection fraction (HFpEF; EF ≥50%) are two major HF subtypes that differ in terms of pathophysiology and etiology, as well as treatment responses [20]. The two phenotypes differ in the proportions with given risk factors. For example, female gender and greater age characterize HFpEF. Comorbidities, key mortality risk factors, neurohormonal and biochemical profile, and progression to structural and functional remodeling differ somewhat between the two phenotypes [21,22,23,24]. For instance, hypertensive heart disease and atrial fibrillation are known to be the risk factors for developing HFpEF. However, these risk factors are key contributors to HFrEF and coronary artery disease. In recent years, several clinical studies have been devoted to identifying additional parameters that could be utilized for HF subtype differentiation. A prospective randomized controlled multicenter trial, TIME-CHF, which included 499 HFrEF and 123 HFpEF patients, found that HFpEF patients were generally older more often females with higher prevalence of hypertension and higher body mass index. Additionally, on initial presentation, a higher percentage of HFpEF patients exhibited edema and had pulmonary crepitations present [25]. In one preclinical left ventricular diastolic dysfunction cohort study, the authors found that HFrEF patients were predominantly male with high percentage of cerebrovascular disease and low baseline of LVEF, while HFpEF patients more often had pulmonary diseases with renal impairment and anemia [26]. A recent study involving a multinational registry of Asian HF patients involving 46 medical centers across Asia observed a similar burden of comorbidity in HFrEF and HFpEF as seen in Western countries. Intriguingly, unlike the West, HF patients in Asia were younger with heterogeneous comorbidities across regions and ethnicities [27]. European recommendations suggested the diagnosis of HFpEF on the basis of the presence of clinical symptoms of heart failure and diastolic dysfunction where EF > 50%. In addition, in the compensated state, chronic HFpEF patients may have normal or near normal plasma natriuretic peptide concentrations, especially in those who are obese or manifest symptoms only upon exertion [28,29]. The diagnosis of non-acute HFpEF remains a challenge as no single diagnostic method is definitive in this clinical setting. This is further complicated by the recent creation of a third HF subtype, mid-range ejection fraction (HFmrEF; 41% ≤ EF ≤ 49 %). The recognition of HFmrEF, previously known as the “grey zone“ with borderline EF, as an emerging HF phenotype has been considered in multiple recent studies [30]. The Chronic Heart Failure Analysis and Registry in Tohoku District 2 (CHART-2) study showed that HFmrEF presents clinical features intermediate between HFrEF and HFpEF and may dynamically transition between these two groups [31]. Apart from LVEF cut-off criteria, both HFmrEF and HFpEF patients require at least one additional criterion of either relevant structural heart disease or diastolic dysfunction; however, with regards to ischemic etiology and drug responses, HFmrEF seems to be similar to HFrEF [32,33,34]. Interestingly, results from several lines of evidence suggested that the ejection fraction of different HF subtypes may change over time and have different clinical outcomes upon drug treatments and prognosis [33], suggesting the underlying pathophysiological mechanism might be interchangeable between subgroups of the different HF subtypes. Nevertheless, the molecular mechanisms underlying transitions between HFrEF, HEmrEF, and HFpEF remain largely unknown. 

The poor prognosis in HF, despite advances in treatment, mandates efforts to enhance management and improve patient outcomes. HF is underpinned by multiple risk factors, comorbidities, and pre-existing conditions which have an adverse impact on cardiac structure and function. The first line of HF drugs were directed towards improving quality of life by attenuating symptoms and slowing down disease progression, as well as reducing morbidity and mortality in HFrEF. However, to date, no HF therapeutics have consistently and permanently halted progression of structural and functional cardiac deterioration. Drugs targeting the renin-angiotensin-aldosterone system (RAAS) including angiotensin-converting enzyme inhibitors (ACEIs) or angiotensin receptor blockers (ARBs), β-adrenergic receptor blockers, and mineralocorticoid receptor antagonists (MRAs) have been shown to ameliorate the symptoms of HF and improve outcomes including HF re-admissions and mortality [10,35]. Recent evidence especially the “PARADIGM” trial, have shown LCZ696, a combination of ARB and neprilysin inhibitor, reduces hospital admissions and lowers mortality in HFrEF patients compared with ACE inhibitor-based therapy [36,37]. However, where tested to date, therapies effective in HFrEF have not reduced mortality in HFpEF. Results from several randomized clinical trials using ACEIs, ARBs, β-blockers, and MRAs have not shown compelling improvement in decreasing the mortality in HFpEF patients. Nevertheless, probable beneficial effects of these drugs in HFpEF patients were observed from different studies. For instance, in the TOPCAT trial, spironolactone (one of the MRAs) was reported to reduce congestion in HFpEF patients [38]. In the CHARM-preserved trial, candesartan (one of the ARBs) was shown to reduce HF admissions in HFpEF patients [39]. Diuretics and acute vasodilator therapy can reduce congestion and provide symptomatic relief of edema and dyspnea in HFpEF patients [20,40]. A recent systematic review and meta-analysis of data from 25 randomized clinical trials suggested that β-blockers provide modest reduction in all-cause (22%) and cardiovascular (25%) mortality in patients with HFpEF [41].

To facilitate the development of next generation HF therapeutics, key knowledge gaps in the underlying mechanisms and pathophysiology of HF must be addressed. The current lack of circulating biomarkers that could provide definitive diagnosis, prognosis, risk stratification, and subtype categorization of HF remains an ongoing challenge. The detailed molecular mechanisms underlying HF pathogenesis and how pathological pathways differ between subtypes and their translation to phenotype-specific therapeutic strategies remain elusive. Unravelling the relationship between biomarkers and HF pathogenesis may shed the light for developing precision medicine for HF in the near future. 

## 4. MicroRNAs, the Small Nucleotides that May Play Big Roles in HF Research

MicroRNAs comprise a cluster of noncoding RNA species that are known to play important roles in modulating the expression of most protein coding genes at the post-transcriptional level [42]. MicroRNAs are first transcribed in the nucleus by RNA polymerase II, subsequently edited and exported to the cytoplasm for further processing into mature microRNA, and finally assembled into argonaut-containing RNA-induced silencing complexes (RISC). In the cytoplasm, by Watson–Crick base pairing, the seed region of microRNA guides the RISC to the 3’ untranslated region (3’UTR) of target gene transcripts and subsequent silencing of gene expression by facilitating the degradation of messenger RNA (mRNA) and/or suppressing the translational machinery [43]. In addition to autonomously regulating gene expression, a growing body of evidence further supports the role of microRNAs in exerting paracrine effects via extracellular vesicles or exosomes and subsequently modulating the biological functions of a targeted organ [44,45,46]. Given the roles of microRNAs in fine-tuning gene expression in cells, and that the circulating microRNA landscape is contingent on active export from cells and/or passive release from damaged or necrotic cells, it is conceivable that the composition of circulating microRNAs may reflect physiological status and may contain valuable pathobiological information of different disease states. MicroRNAs, particularly circulating microRNAs, have been found to be highly stable with long half-lives under a controlled environment [47,48]. Accumulating evidence from studies across broad biomedical disciplines suggests the involvement of microRNAs, and their potential applications as diagnostic as well as prognostic biomarkers in various human diseases including cancer [49,50,51], neurodegenerative diseases [52,53,54,55], fertility disorders [56,57], and metabolic disorders [58,59]. For several years, our research effort has been devoted to discovering potential applications of microRNAs in disease diagnosis, as well as elucidating the role of dysregulated microRNAs in cardiovascular pathogenesis [60,61,62,63,64]. The involvement of specific microRNAs in different cardiovascular pathological pathways has been examined using functional analyses and different disease models [65], and the dysregulation of circulating microRNAs offers candidate biomarkers for heart failure [43].

## 5. MicroRNA Profiling in HFrEF and HFpEF

With increasing understanding of the characteristic differences between HFrEF and HFpEF, there has been active interest in finding signature microRNA profiles to facilitate HF subtype categorization. Ellis et al. pioneered early work on HFrEF and HFpEF microRNA profiling and reported a set of differentially expressed microRNAs in a dyspnea cohort comprising 16 HFrEF and 16 HFpEF with high NT-proBNP plasma concentrations [66]. However, this preliminary set of microRNAs could not be validated in a second cohort. The authors suggested the discrepancy might reflect differences in cohort recruitment criteria and small sample sizes. In 2015, Watson et al. and our group concurrently published signature microRNAs that differentiated HFrEF from HFpEF using larger HF cohorts. Both groups found that the diagnostic performance could be enhanced by combining the levels of microRNA signatures with NT-proBNP/BNP [62,67]. In 2018, Chen et al. reported the identification of two highly upregulated microRNAs, miR-3135b and miR-3908, in HFpEF, and highlighted their potential as markers differentiating HFrEF from HFpEF. However, the diagnostic performance of these two microRNAs was not examined [68]. The differentially expressed microRNAs in HFrEF and HFpEF identified in the four aforementioned cohort studies is summarized in Table 1.

**Figure 1:**
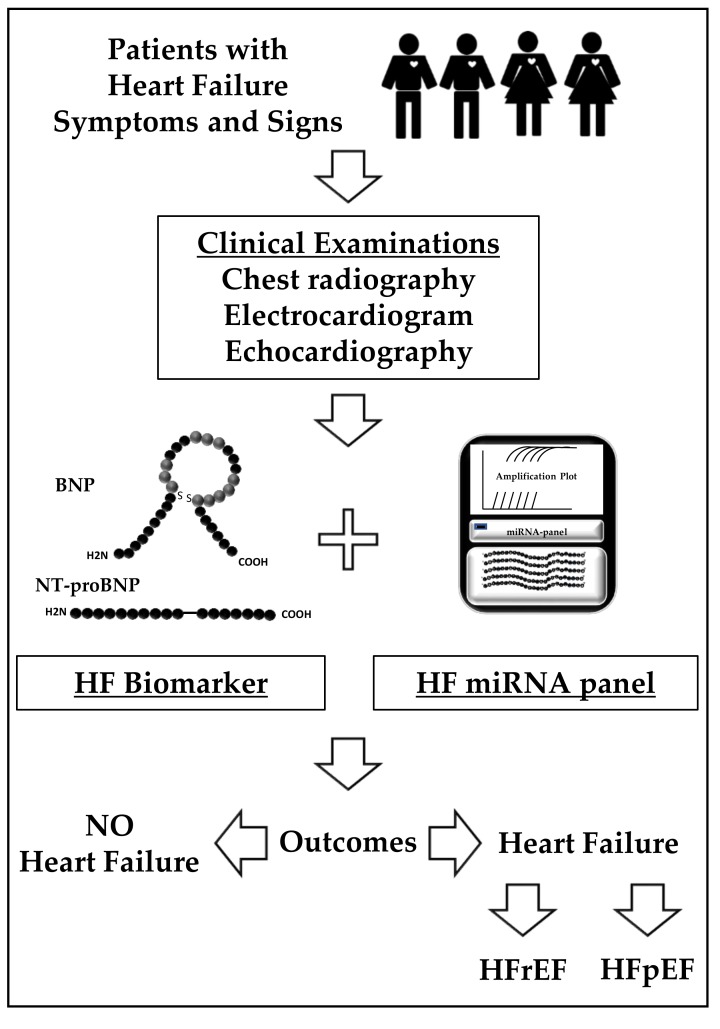
Proposed roadmap of diagnosis of heart failure (HF) and HF subtypes based on clinical diagnosis using typical HF symptoms and signs, followed by combination of the natriuretic peptides and microRNA panels.

It is noteworthy that each study reported a unique set of differentially dysregulated microRNAs and no overlap of microRNAs was observed across all four cohort studies. This discrepancy is not totally unexpected and may be attributed to the heterogenous comorbidities of HF and sample size of cohorts, as well as the different criteria used for patient enrollment. Profiling reported in the five studies highlighted in Table 1 were obtained using different platforms involving SYBR-based single-plex qPCR assay, miRCURY LNA microRNA microarrays, and miRCURY LNA miRNA miRNome PCR Panel kit. Each group arguably selected the most suitable platforms for their studies based on assay sensitivity, extent of microRNA entities coverage, commercial availability, and accessibility of related hybridization and chip-scanning facilities. Therefore, the discrepancies observed among the various studies may also be contributed by the different microRNA profiling platforms applied in each study. In an effort to minimize the contribution of batch-to-batch discrepancy, a recent large HF cohort study (1657 participants including 850 with HF) comprising patients from both Singapore and New Zealand used a quantitative reverse transcription PCR-based method for profiling the microRNA landscape in health and HF [63]. The authors reported that 41 of the 203 preselected cardiovascular-related microRNAs were found to be differentially expressed between HF patients and controls. Notably, the trends of 12 dysregulated microRNAs were consistent with previous cohort studies from our and others’ works. Furthermore, biomarker panels of six or more microRNAs demonstrated higher discriminative power for distinguishing HFpEF from HFrEF compared to plasma NT-proBNP levels alone, while coupling multi-microRNAs panels with NT-proBNP further elevated the discriminative power. Results from our latest cohort study demonstrated for the first time the strength of coupling circulating microRNA panels with NT-proBNP to classify HFrEF from HFpEF. Importantly, the discovery microRNA panels were verified in two independent cohorts from Han and Caucasian populations, suggesting that common HF-related microRNAs exist in different ethnicities with heterogenous comorbidities. Furthermore, our study clearly demonstrated that carefully defined clinical criteria for cohort enrollment is one of the critical steps for studying complex diseases like HF. The discriminative power of the microRNA panels in non-acute HF would provide useful preliminary screening information for the clinicians prior to referring patients for further specialized investigations such as echocardiographic analyses for a final diagnosis of HF and fuller phenotyping. Further analyses of discriminative HF-related microRNAs panels revealed that these could be further delineated to differentiate HFpEF from HFrEF especially where clinical signs and symptoms are ambivalent so that respective HF treatments could be prescribed in a timely fashion. The proposed application of the microRNA-based biomarker panel in HF diagnosis is illustrated in Figure 1.

**Figure 2:**
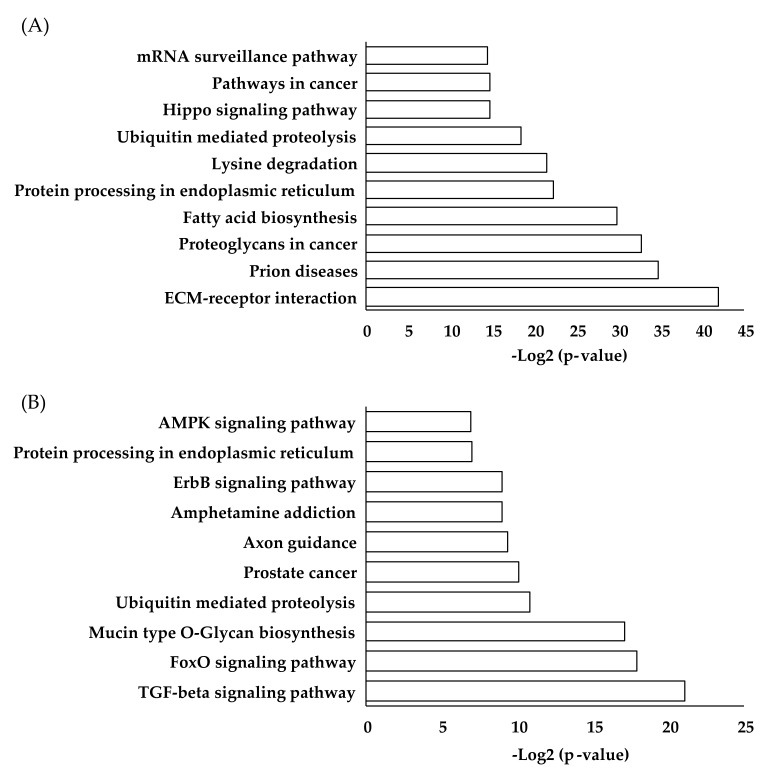
Pathway analyses using DIANA-mirPath V3. Kyoto Encyclopedia of Genes and Genomes (KEGG) pathways regulated by the eight microRNAs (hsa-miR-193a-5p, hsa-miR-30a-5p, hsa-miR-106a-5p, hsa-miR-191-5p, hsa-miR-486-5p, hsa-miR-181a-2-3p, hsa-miR-660-5p, and hsa-miR-199b-5p) that could be used for distinguishing HFrEF from HFpEF. (**A**) Pathway analysis of DIANA-TarBase experimentally validated targets, and (**B**) DIANA-microT-CDs predicted microRNA-mRNA targets showing the top significantly represented biological pathways.

In addition to potential diagnostic applications, the discovery of microRNA clusters that are differentially expressed in HFrEF and HFpEF patients provides an approach to delineate the different pathobiological pathways underlying HFrEF and HFrEF. Figure 2 shows the putative biological pathway affected by a panel of eight HFpEF-related microRNAs (hsa-miR-193a-5p, hsa-miR-30a-5p, hsa-miR-106a-5p, hsa-miR-191-5p, hsa-miR-486-5p, hsa-miR-181a-2-3p, hsa-miR-660-5p, and hsa-miR-199b-5p) as revealed by DIANA-miRPath v3.0 (http://www.microrna.gr/miRPathv3), an online pathway analysis software [69]. Based on analysis of these experimentally validated targets (TarBase), the two most relevant pathways that may contribute to the pathophysiological difference between HFrEF and HFpEF involve extracellular matrix (ECM)-receptor and fatty acid biosynthesis. These findings are corroborated by previous reports of possible pathophysiological involvement of profibrotic processes in HFpEF and further supported by elevations of profibrotic markers, soluble form of interleukin 1 receptor like 1 (IL1RL1, also known as ST2) and galectin-3 (GAL3), as well as the reduction of matrix metalloproteinase-2 (MMP2), an enzyme involved in ECM destruction in HF [70]. The signaling cascades of GAL3 in trans-differentiation of fibroblasts to myoblasts, and the involvement of soluble ST2 in profibrotic signaling as well as the function of MMP2 in collagen degradation have been well established. Future work should be geared towards probing the regulatory roles of these eight signature microRNAs as possible key molecules in profibrotic signaling and exploring the putative cross talk between different signaling cascades including microRNAs. 

Changes in plasma concentrations of free fatty acids across three different HF subtypes have been reported previously [47]. Results from a clinical cohort revealed that patients with HFrEF tend to have higher free fatty acids than those with HFmrEF and HFpEF, suggesting differences in the mechanism of impaired energetics between different HF subtypes. Omega-3 polyunsaturated fatty acids (ω3-PUFAs), eicosapentaenoic acid (EPA), and docosahexaenoic acid (DHA) are important fatty acids in cardiovascular homeostasis. The ω3-PUFAs and EPA have been shown to attenuate HF remodeling by activation of cyclic guanosine monophosphate (cGMP) signaling, and by antagonizing transforming growth factor beta 1 (TGF-β1) signaling in cardiac fibroblast in a pressure overload mouse model [71,72]. Impairment of cardiac fatty acid oxidation (FAO) leads to diastolic dysfunction in angiotensin II infused adult mice, and this pathological effect is reversed when FAO is restored [73]. Thus, the role of the eight signature microRNA in fatty acid biosynthesis provide a potential therapeutic avenue for future exploration. 

Interestingly, predicted targets analysis (microT-CDs) revealed that transforming growth factor beta (TGF-β) signaling and Forkhead box O (FoxO) transcription factors signaling are among the top pathways predicted to be affected by the eight HF signature microRNAs. TGF-β signaling is known to play pivotal roles in the pathogenesis of cardiac remodeling and fibrosis. The putative roles of microRNAs in TGF-β signaling have been reported and tested in various diseased models. For instances, miR-101a was shown to target the TGF-β1 receptor, subsequently attenuating fibrogenesis and improving cardiac function in a rodent transverse aortic constriction (TAC) model [74]. FoxOs are known to transduce a wide range of extracellular signals and play important roles in controlling cellular fate such as cell survival and cell cycle progression. Ablation of FoxO1 in endothelial cells inhibited basal and vascular endothelial growth factor-mediated serine/threonine kinase 1 (AKT) signaling and subsequently disrupted vascular homeostasis [75]. The critical role of FoxO in cardiomyopathy was further supported by the recovery of cardiac function in an induced diabetic mouse model without FoxO [76]. The involvement of the eight signature microRNAs in TGF-β and FoxO signaling remains largely unknown and further mechanistic studies focused on these pathways could bring new insights into the pathobiological differences between HFrEF and HFpEF. Interestingly, as shown in Figure 2, pathways that involve ubiquitin-mediated proteolysis and protein processing in endoplasmic reticulum appear in both Tarbase and microT-CDs analyses, indicating the possible involvement of these signature microRNAs in post-translational modification, thus charting further possible new territory for future investigations.

## 6. Future Perspectives and Follow-Up Study

The discovery of microRNAs and their roles in post-transcriptional modification have led to further advances in understanding the complexity of gene regulation. However, translating findings of distinct circulating microRNA compositions and their pathophysiological implications in different disease states to decipher underlying mechanisms with a view towards therapeutic exploitation remains challenging. 

MicroRNA-3’UTR interactions do not require perfect Watson–Crick pairing and this significantly increases the functional flexibility of microRNA. It is now known that one microRNA can have multiple 3’UTR targets, and one 3’UTR can be recognized by more than one microRNA so that biological homeostasis could be tightly controlled in the normal state while dynamic biochemical responses could also be triggered upon stimulation. Although it has been established that microRNAs play important roles in post-transcriptional modification, scientists have yet to fully understand the operating rhythm of microRNAs. The traditional approach of deciphering the regulatory effect of one microRNA on one gene is manifestly inadequate in bringing together the influence of the microRNA landscape on gene expression regulatory networks. Dysregulated microRNAs can be viewed as functional clusters that variously contribute to HF pathogenesis. This concept was supported by our previous study where data from microRNA/3’UTR binding and gain-of-function assays demonstrated putative regulatory effects of HF-related microRNAs upon key molecules involved in neurohormonal signaling [64]. 

Given the potential application of signature microRNAs in HF diagnosis, further independent verification of our findings by other reputable investigators is needed. Furthermore, the diagnostic performance of microRNAs may require further refinement according to comorbidities and clinical background. Further studies to clarify the association between HF-related microRNAs and pathogenesis will be essential to translate these epigenetic alterations into therapeutic applications. It is conceivable that linear algorithms will not be adequate to describe the interaction networks between transactivation and post-transcriptional regulation of microRNAs with their target genes. Hence, large scale international multi-disciplinary collaborative research integrating expertise in biomedicine, clinical practice, bioinformatics, artificial intelligence, and machine learning will be pivotal to advancing progress in HF research. 

## Figures and Tables

**Table 1:** Summary of reported circulating microRNA entities that differentially expressed in HFrEF and HFpEF patients.

Discovery Cohort	Validation Cohort	microRNAs Identified	Diagnostic Performance	References
16 HFrEF and 16 HFpEF (Plasma/qPCR)	22 HFrEF and 22 HFpEF (Plasma/qPCR)	miR-342-3p, miR-199a-3p, miR-150, miR-29a, miR-2110, miR-27b, miR-940 and miR-23a	Not available	Ellis KL [66]
39 HFrEF and 19 HFpEF (Whole blood /miRCURY LNA^TM^ array)	30 HFrEF and 30HFpEF (Plasma/qPCR)	miR-125a-5p, miR-190a, miR-550a-5p and miR-638	Individual markers AUC of 0.58-0.80 Combining NT-proBNP AUC of 0.79-1.00	Wong LL [62]
15 HFrEF and 15 HFpEF (Serum/ Taqman array Human MicroRNA card set v3)	75 HFrEF and 75 HFpEF (Serum/qPCR)	miR-146a, miR-221, miR-328, miR-375 and miR-30c	Individual markers AUC of 0.52-0.75 Combining NT-proBNP AUC of 0.67-0.86	Watson CJ [67]
13 HF (Plasma /miRCURY LNA^TM^ array)	18 HFrEF and 14 HFpEF (Plasma/qPCR)	miR-3135b, miR-3908 and miR-5571-5p	miR-3135b and miR-3908 were upregulated in HFpEF vs HFrEF	Chen F [68]
180 HFrEF and 158 HFpEF (Plasma/qPCR)	Validation 1: 116 HFrEF and 72 HFpEF Validation 2: 145 HFrEF and 179 HFpEF (Plasma/qPCR)	41 miRs dysregulated in discovery cohort; 8-miRNA panels: miR-193a-5p, miR-30a-5p, miR-106a-5p, miR-191-5p, miR-486-5p, miR-181a-2-3p, miR-660-5p and miR-199b-5p	8-miRNA markers AUC of 0.65-0.81 Combining NT-proBNP AUC of 0.72-0.87	Wong LL [63]
